# Supplementary material for: EnzML: multi-label prediction of enzyme classes using InterPro signatures
Source: BMC Bioinformatics. 2012 Apr 25;13:61. doi: 10.1186/1471-2105-13-61 (PMC3483700; doi:10.1186/1471-2105-13-61)
Supplement: Addtional file 5 — The Java code to format the data files, evaluate and predict. The file enzml_java_code.tar.gz contains the Java code used to format database data to ARFF and XML formats, to execute cross and train-test (jackknife) evaluations and to record evaluation results to database. More information is included in the readme.txt file and the Javadoc files. The code can be used with a MySQL database. To use a different database software, other JDBC drivers might be required. [file 1471-2105-13-61-S5.gz › java_code/enzml2011/doc/uk/ac/ed/inf/taweka/class-use/DataSetManager.html]

Uses of Class uk.ac.ed.inf.taweka.DataSetManager


---


|  |  |  |
| --- | --- | --- |
| **Overview** \| **Package** \| **Class** \| **Use** \| **Tree** \| **Deprecated** \| **Index** \| **Help** | |  |
| PREV   NEXT | **FRAMES**    **NO FRAMES**     **All Classes** |  |


---


## **Uses of Class uk.ac.ed.inf.taweka.DataSetManager**

| Packages that use DataSetManager | |
| --- | --- |
| **uk.ac.ed.inf.taweka** |  |
| **uk.ac.ed.inf.taweka.mulan** |  |
| **uk.ac.ed.inf.taweka.test** |  |

Uses of DataSetManager in uk.ac.ed.inf.taweka

| Methods in uk.ac.ed.inf.taweka that return DataSetManager | |
| --- | --- |
| `DataSetManager` | `DataSetDbLoader.getDataSetManager()` |
| `DataSetManager` | `ArffGenerator.getDataSetManager()` |
| `DataSetManager` | `DataSetWriter.getDataSetManager()` |
| `DataSetManager` | `DataSetGenerator.getManager()` |
| `DataSetManager` | `DataSetChecker.getManager()` |

Constructors in uk.ac.ed.inf.taweka with parameters of type DataSetManager

`DataSetChecker(DataSetManager manager)`

`DataSetDbLoader(DataSetManager manager)`

`DataSetGenerator(DataSetManager manager,
uk.ac.ed.inf.utils.OneToManyMap instanceAttributeMap,
uk.ac.ed.inf.utils.OneToManyMap instanceClassMap)`

`DataSetWriter(DataSetManager manager)`

Uses of DataSetManager in uk.ac.ed.inf.taweka.mulan

| Subclasses of DataSetManager in uk.ac.ed.inf.taweka.mulan | |
| --- | --- |
| `class` | `MulanDataSetManager`             This class generates a data set (instances, attributes, classes) in Mulan style (for multi-label machine learning). |

Uses of DataSetManager in uk.ac.ed.inf.taweka.test

| Methods in uk.ac.ed.inf.taweka.test that return DataSetManager | |
| --- | --- |
| `static DataSetManager` | `DataSetManagerTest.getDataSetManager()` |
| `static DataSetManager` | `DataSetManagerTest.getDataSetWithNoXmlProperty()` |

---


|  |  |  |
| --- | --- | --- |
| **Overview** \| **Package** \| **Class** \| **Use** \| **Tree** \| **Deprecated** \| **Index** \| **Help** | |  |
| PREV   NEXT | **FRAMES**    **NO FRAMES**     **All Classes** |  |


---
